# Survival of HIV-Infected Adolescents on Antiretroviral Therapy in Uganda: Findings from a Nationally Representative Cohort in Uganda

**DOI:** 10.1371/journal.pone.0019261

**Published:** 2011-04-29

**Authors:** Celestin Bakanda, Josephine Birungi, Robert Mwesigwa, Jean B. Nachega, Keith Chan, Alexis Palmer, Nathan Ford, Edward J. Mills

**Affiliations:** 1 The AIDS Support Organization (TASO), Kampala, Uganda; 2 Department of Medicine and Centre for Infectious Diseases, Stellenbosch University, Faculty of Health Sciences, Cape Town, South Africa; 3 British Columbia Centre for Excellence in HIV/AIDS, Vancouver, British Columbia, Canada; 4 Centre for Infectious Disease Epidemiology and Research, University of Cape Town, Cape Town, South Africa; 5 Faculty of Health Sciences, University of Ottawa, Ottawa, Ontario, Canada; Karolinska Institute, Sweden

## Abstract

**Background:**

Adolescents have been identified as a high-risk group for poor adherence to and defaulting from combination antiretroviral therapy (cART) care. However, data on outcomes for adolescents on cART in resource-limited settings remain scarce.

**Methods:**

We developed an observational study of patients who started cART at The AIDS Service Organization (TASO) in Uganda between 2004 and 2009. Age was stratified into three groups: children (≤10 years), adolescents (11–19 years), and adults (≥20 years). Kaplan-Meier survival curves were generated to describe time to mortality and loss to follow-up, and Cox regression used to model associations between age and mortality and loss to follow-up. To address loss to follow up, we applied a weighted analysis that assumes 50% of lost patients had died.

**Findings:**

A total of 23,367 patients were included in this analysis, including 810 (3.5%) children, 575 (2.5%) adolescents, and 21 982 (94.0%) adults. A lower percentage of children (5.4%) died during their cART treatment compared to adolescents (8.5%) and adults (10%). After adjusting for confounding, other features predicted mortality than age alone. Mortality was higher among males (*p*<0.001), patients with a low initial CD4 cell count (*p*<0.001), patients with advanced WHO clinical disease stage (*p*<0.001), and shorter duration of time receiving cART (*p*<0.001). The crude mortality rate was lower for children (22.8 per 1000 person-years; 95% CI: 16.1, 29.5), than adolescents (36.5 per 1000 person-years; 95% CI: 26.3, 46.8) and adults (37.5 per 1000 person-years; 95% CI: 35.9, 39.1).

**Interpretation:**

This study is the largest assessment of adolescents receiving cART in Africa. Adolescents did not have cART mortality outcomes different from adults or children.

## Introduction

Programmatic evaluations of HIV/AIDS in resource-limited settings have historically focused on adult and child populations [1], [2] There is growing appreciation, however, that other age groups pose a particular challenge to the provision of combination antiretroviral therapy (cART). For instance, the number adolescents on cART continues to increase [3]. This is largely a reflection of successful treatment of perinatally-infected children, infections during early adolescence, and the expansion of access to cART worldwide [3] Globally, in 2008, over 40% of all new reported HIV infections occurred in young people ages 15–24 [4]. A 2009 study from Southern Africa, Ferrand et al predicts a substantial epidemic among perinatally-infected adolescents despite previous assertions that few of these children would reach adolescence [5]. As these children mature and enter adolescence, it is important that appropriate services are available to counsel youth about sexual safety, adherence to ART and reproductive choices in order to prevent further horizontal transmission.

Adolescence can be a confusing time for youth, especially those living with a chronic and often stigmatized disease. A number of challenges have been identified that may compromise positive outcomes of care for adolescents. They may be particularly rebellious, may not have caregivers unlike younger children, and there many be challenges associated with puberty and disease [6]. The few published studies examining outcomes of care among adolescents on cART report that cART access and adherence is lower in adolescents than in adults [7]–[12]. Nachega and colleagues published the only study reporting adolescent clinical outcomes in Africa.^13^ In this relatively small sample, (n = 154), the authors reported significantly worse virological suppression in adolescents versus adults in a cohort of patients from nine southern African countries receiving privately purchased cart [13]. No specific data on reasons of non-adherence were documented in that study and authors speculated on possible factors of non-adherence to cART and social (stigma/discrimination; social support) or structural (cost, access to care) issues in their privately managed AIDS Care pilot study population [13].

Not all settings in Africa are alike, neither can we expect that outcomes in a privately purchased cART program will be similar to a publicly funded one. This study compares survival and loss to follow-up of adolescents to children and adults using a large dataset from a nationally representative cohort of HIV patients receiving free cART in Uganda.

## Methods

### Ethics statement

This study received ethical approval from TASO Administrative Research Board, a Uganda National Science and Technology Council approved board, and from University of British Columbia. Informed consent was not required as this was routinely collected operational data and the institutional review boards waived the need for consent.

### Programme

The AIDS Support Organization (TASO) is the largest and oldest national non-governmental AIDS service organization in Africa. Founded in 1987, TASO currently provides psychosocial support, clinical care, and cART free of charge to people with HIV in Uganda. TASO started prescribing cART in 2004 and now provides treatment to approximately 24 000 patients throughout 11 clinical sites in Uganda. Criteria used for initiation of cART include World Health Organization (WHO) clinical disease stage III or IV or a CD4 cell count below 250 cells/mm^3^ [14]. Patients starting cART typically receive an initiation regimen of non-nucleoside reverse transcriptase inhibitor with first-line treatment comprising nevirapine, lamivudine and stavudine and boosted lopinavir, didanosine and zidovudine as second-line [15], [16].

### Data Collection

This study is based on data collected routinely for clinical monitoring and evaluation purposes at TASO. Clinicians complete standardized patient forms detailing patient demographics, clinical, psychosocial, and drug utilization data at each patient visit. These data are then entered into the TASO data collection database at each site by trained data capturers. All data are anonymized using a unique, confidential identification number.

Clinic staff, nurses and clinical officers or physicians offer adherence monitoring and clinical support. For community-based recipients of care, a field monitoring team equipped with motorcycles is responsible for patient adherence, social support, and follow-up. This team, which includes medical attendants who conduct HIV testing, adherence counselling, clinical observation, and provide cART to patients, visits patients who fail to show for any appointment for three months or longer and patients who have requested home-based care.

The present study includes all patients who initiated cART since the start of the programme (2004), irrespective of age. For each patient, the following baseline information was recorded: age at start of cART (years), gender (male, female), exact baseline CD4 cell count, WHO clinical disease stage, loss to follow-up status, date last seen for care, and where applicable, date of death.

### Data Analysis

The cohort was stratified into three age groups: children (age ≤10 years), adolescents (age 11–19 years), and adults (age ≥20 years). These age definitions have been used before by Nachega et al. [13]. Baseline characteristics between age groups were assessed for skew and compared using analysis of variance. We used Weibull hazards to model the associations between age and risk of mortality and loss to follow-up, including gender and baseline CD4 cell count as potential confounders [17]. Hazard proportionality was assessed by analysis of scaled Schoenfeld residuals. Kaplan-Meier survival curves were generated to describe time to mortality and loss to follow-up for the three age groups, with survival curves compared using the log-rank test. Survival times were expressed in months. We assessed the potential misclassification of mortality among those lost to follow-up by assuming that 50% of the patients lost to follow-up had died. We weighted this assumption according to individuals with lower baseline CD4 status using a random sequence generator. This figure of 50% mortality among defaulters is consistent with evaluations examining the extent of attrition associated with mortality [18]. All significance tests were two-sided with a *p*-value of <0.05 considered significant. All analyses were conducted using SAS version 8 (SAS Institute, Cary, NC).

## Results

A total of 23,367 patients were included in this analysis. There were 810 (3.5%) children, 575 (2.5%) adolescents, and 21,982 (94.0%) adults at baseline. Follow-up time differed between the age groups. Adults were followed for a median of 32 months (IQR: 19, 45), and children and adolescents were followed for 28 months (IQR: 18, 38) and 28 months (IQR: 17, 38), respectively (*p*<0.001).

Table 1 shows the demographic and clinical characteristics of patients by age group. The majority of patients overall were female, and there was a significantly higher proportion of females than males in the adolescent and adult age groups (*p*<0.001). Baseline CD4 at the time of cART initiation was significantly different across the age groups (*p*<0.001): child patients started cART at a median CD4 count of 238 cells/µl (IQR: 91, 490), adolescent patients started at a median of 131 cells/µl, (IQR: 46, 213) and adult patients started at a median of 142 cells/µl (IQR: 71, 206). A lower percentage of children (5.4%) died during their cART treatment compared to adolescents (8.5%) and adults (10%). There were also no significant differences in WHO clinical disease stage and loss to follow-up between the 3 age groups.

**Table 1:** Characteristics of study participants by age group.

Variable	Child(≤10 years)	Adolescent(11–19 years)	Adult(≥20 years)	*p*-value
***Gender***				
Female	411 (50.7%)	361 (62.8%)	15,259 (69.4%)	<0.001
Male	399 (49.3%)	214 (37.2%)	6,723 (30.6%)	
***CD4 cell count (cells/mm^3^)***				
<50	105 (16.1%)	121 (26.4%)	3,364 (18.5%)	<0.001
50–99	75 (11.5%)	69 (15%)	2,900 (15.9%)	
100–199	99 (15.2%)	137 (29.8%)	6,926 (38.0%)	
200–299	107 (16.4%)	64 (13.9%)	2,985 (16.4%)	
≥300	267 (40.9%)	68 (14.8%)	2,047 (11.2%)	
***WHO Stage***				
Stage I	7 (1.3%)	11 (2.9%)	459 (3.2%)	0.192
Stage II	301 (56.3%)	218 (58.4%)	7,861 (54.5%)	
Stage III	179 (33.5%)	115 (30.8%)	4,908 (34.0%)	
Stage IV	48 (9.0%)	29 (7.8%)	1,201 (8.3%)	
***Death***				
No	773 (95.4%)	536 (93.2%)	20,510 (93.3%)	0.057
Yes	37 (4.6%)	39 (6.8%)	1,472 (6.7%)	
***Lost to follow-up***				
No	771 (95.2%)	533 (92.7%)	20,573 (93.6%)	0.124
Yes	39 (4.8%)	42 (7.3%)	1,409 (6.4%)	
***Year of first therapy***				
Median (IQR)	2007 (2006–2008)	2007 (2006–2008)	2007 (2005–2007)	<0.001

Overall, mortality was higher among males (*p*<0.001), patients with a CD4 cell count lower than 100 cells/mm^3^ at cART initiation (*p*<0.001), patients with a WHO clinical disease stage of III or IV at cART initiation (*p*<0.001), and patients who started cART at an earlier time point (*p*<0.001). The crude mortality rate was lower for children (22.8 per 1000 person-years; 95% CI: 16.1, 29.5), than adolescents (36.5 per 1000 person-years; 95% CI: 26.3, 46.8) and adults (37.5 per 1000 person-years; 95% CI: 35.9, 39.1).

**Figure 1:**
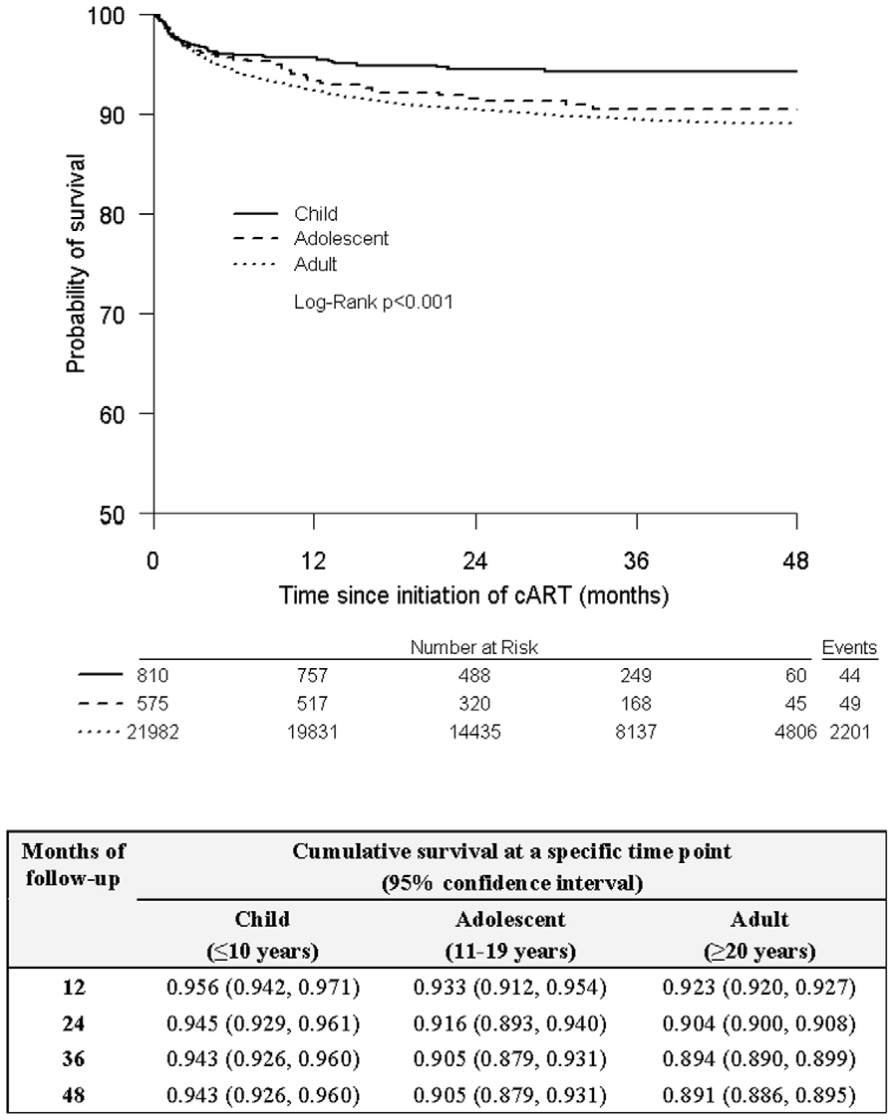
Kaplan-Meier product limit estimates for the probability of survival.

Figure 1 shows the Kaplan-Meier curve for time to mortality. At 12 months, the probability of survival was 0.956 (95% CI: 0.942, 0.971) for children, 0.933 (95% CI: 0.912, 0.954) for adolescents, and 0.923 (95% CI: 0.920, 0.927) for adults. By 48 months, this was 0.943 (95% CI: 0.926, 0.960) for children, 0.905 (95% CI: 0.879, 0.931) for adolescents, and 0.891 (95% CI: 0.886, 0.895) for adults. The *p*-value of the log-rank test was <0.001, suggesting an initial difference in the survival distributions between the age groups.

Table 2 summarizes the unadjusted and adjusted Weibull hazards model for mortality. After adjusting for gender, baseline CD4 cell count, and year of therapy initiation, there was no difference between adolescents and children (aHR: 0.74; 95% CI: 0.47, 1.14) or adolescents and adults (aHR: 1.07; 95% CI: 0.78, 1.46).

**Table 2:** Multivariate analysis of risk of death.

Variable	Unadjusted(95% confidence interval)	*p*-value	Adjusted(95% confidence interval)	*p*-value
***Age group***				
Adolescent (11–19 years)	1.00		1.00	
Child (≤10 years)	0.63 (0.42,0.94)	0.025	0.74 (0.47,1.14)	0.174
Adult (≥20 years)	1.10 (0.83,1.46)	0.501	1.07 (0.78,1.46)	0.683
***Gender***				
Male *versus* Female	1.50 (1.38,1.63)	<0.001	1.41 (1.29,1.55)	<0.001
***CD4 cell count (cells/mm^3^)***				
<50	1.00		1.00	
50–99	0.60 (0.53,0.68)	<0.001	0.60 (0.52,0.68)	<0.001
100–199	0.40 (0.36,0.45)	<0.001	0.41 (0.36,0.45)	<0.001
200–299	0.32 (0.27,0.38)	<0.001	0.35 (0.30,0.42)	<0.001
≥300	0.28 (0.23,0.34)	<0.001	0.33 (0.27,0.41)	<0.001
***WHO Stage***				
Stage I or II	1.00			
Stage III	1.87 (1.67,2.10)	<0.001		
Stage IV	3.64 (3.14,4.23)	<0.001		
***Year of first therapy***				
Per 1 year increase	0.82 (0.80,0.85)	<0.001	0.85 (0.82,0.89)	<0.001

Note: 50% of those lost to follow-up were assumed dead.

**Figure 2:**
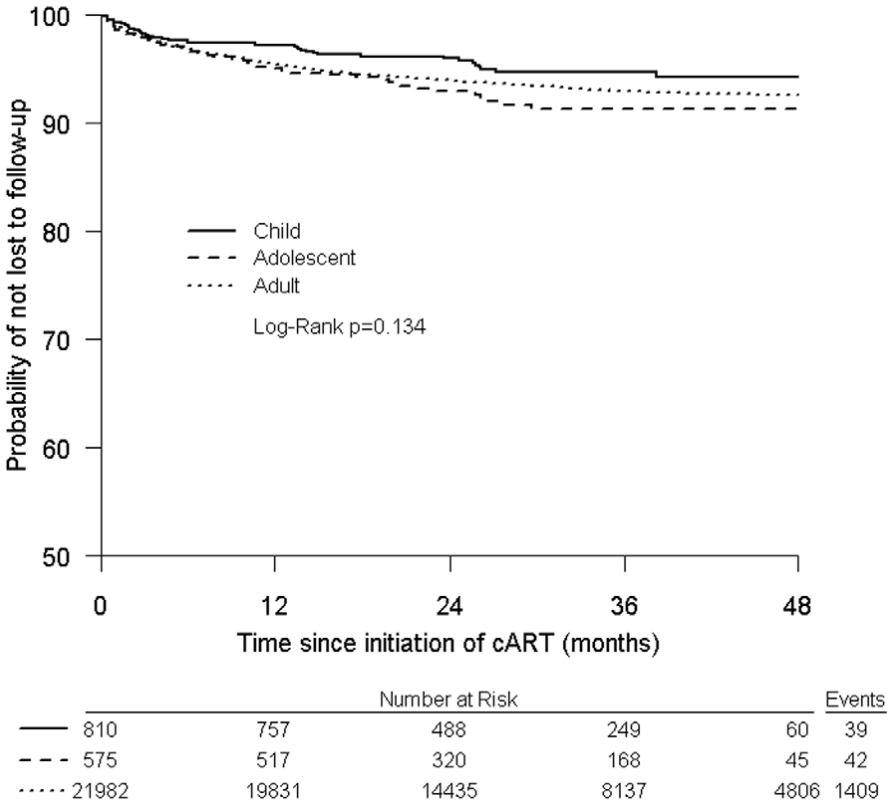
Kaplan-Meier product limit estimates for time to loss to follow-up.

Figure 2 displays the Kaplan-Meier curve for the sensitivity analysis of time to loss to follow-up. The *p*-value of the log-rank test was 0.134, suggesting there is no difference in the distributions of age for loss to follow-up.

## Discussion

This study is the largest assessment of clinical outcomes among adolescents receiving cART in Africa. In this study, crude adolescent mortality was significantly different from child patients, yet, loss to follow-up was not. After adjusting for explanatory variables, we did not demonstrate a significance difference in either mortality or loss to follow up across groups.

The recent report from southern Africa by Nachega et al. found that adolescents have worse outcomes compared to their adult counterparts in terms of virologic suppression and adherence [13], but did not explore survival. Nachega and colleagues examined adherence to cART as a possible predictor of virological suppression in adolescents compared to adults receiving cART from a private provider managed AIDS care programme in Southern Africa and found an increased rate of virological failure among adolescents when compared to adults (Hazard Ratio 3.03, 95% CI: 1.31–3.13) [13]. Possible explanations for increased virological failure in adolescents include poorer pharmacy refill adherence than adults and lack of social support. It is likely that outcomes such as mortality and adherence are influenced by region and programme level factors (e.g. private versus public sector), which merit further research.

As most adolescents would be infected at birth, many individuals would have died before achieving adolescence. As the number of adolescents enrolled in treatment grows and patient live longer, the question of adherence and retention will become increasingly important for health care practitioners. Often adolescents fall through the cracks between pediatric care and adult care. Specialized adolescent health care clinics providing counseling, testing, and treatment have been developed in countries like South Africa to meet the needs of HIV-positive and at-risk youth [19].

As with almost all assessments of mortality in African cohorts, we found that male gender was independently predictive of mortality. This finding is inconsistent with advocacy for increased attention to female issues and we hope that the medical and advocacy communities can promote an evidence-based strategy to responding to local epidemics. In our experience, and others, male patients are typically late to receive cART, have more advanced illness, and have worse clinical outcomes [20].

As with any study of this nature, there were several limitations. Loss to follow-up may have led to a misclassification of mortality as loss to follow up. TASO uses active retention strategies to locate patients who do not attend their scheduled appointments, thus reducing degree of lost to follow-up. We also attempted to overcome the issue of loss to follow-up in the present study with the use of our assumption that 50% of those lost had died 50% [18]. Although it was not possible to include the primary causes of death in this study. It is likely that the primary causes of death for adolescent patients differ than other age groups.

It should also be noted that CD4 cell count data at cART initiation, was not complete. The lack of complete CD4 cell counts is a reflection of the diverse settings in which TASO works in Uganda. This problem is also common in other resource-constrained settings [21]. Additionally, routine patient data on HIV viral load or antiretroviral resistance testing is not available in our setting. Therefore, we cannot be sure of the number of treatment failures and determinants. Finally, since this is an observational study, no conclusions about causality can be made. As in all observational cohort studies, unmeasured differences may exist among in the population under study.

Strengths of the study include the large sample size and long-term follow-up. The cohort includes patients receiving care throughout Uganda, and thus captures a wide range of differing patient experiences based on regional variation. Furthermore, the use of active retention to reduce loss to follow-up has resulted in higher patient retention rates than similar cohort studies.

In conclusion, our study confirms earlier assertions that providing cART to adolescent patients is a complex undertaking. Adolescents have been overlooked in the literature and also in programming. They have unique needs that require tailored services and targeted research. As this population becomes increasingly important in the epidemic, further investigation into the causes of loss to follow up and mortality are needed for this subgroup of patients in order to design and evaluate supportive strategies for this vulnerable population.
